# Coated cysteamine, a potential feed additive for ruminants — An updated review

**DOI:** 10.5713/ab.23.0245

**Published:** 2023-11-01

**Authors:** Muhammad Umar Yaqoob, Jia Hou, Li Zhe, Yingying Qi, Peng Wu, Xiangde Zhu, Xiaoli Cao, Zhefeng Li

**Affiliations:** 1 Provincial Key Agricultural Enterprise Research Institute of King Techina, Hangzhou King Techina Feed Co., Ltd., Hangzhou 311107, China; 2 College of Animal Science, Zhejiang University, Hangzhou 310058, China

**Keywords:** Anti-oxidant, Cysteamine, Growth Performance, Rumen Fermentation, Rumen Microbiota

## Abstract

For sustainable development, better performance, and less gas pollution during rumen fermentation, there is a need to find a green and safe feed additive for ruminants. Cysteamine (CS) is a biological compound naturally produced in mammalian cells. It is widely used as a growth promoter in ruminants because of its ability to control hormone secretions. It mainly controls the circulating concentration of somatostatin and enhances growth hormone production, leading to improved growth performance. CS modulates the rumen fermentation process in a way beneficial for the animals and environment, leading to less methane production and nutrients loss. Another beneficial effect of using CS is that it improves the availability of nutrients to the animals and enhances their absorption. CS also works as an antioxidant and protects the cells from oxidative damage. In addition, CS has no adverse effects on bacterial and fungal alpha diversity in ruminants. Dietary supplementation of CS enhances the population of beneficial microorganisms. Still, no data is available on the use of CS on reproductive performance in ruminants, so there is a need to evaluate the effects of using CS in breeding animals for an extended period. In this review, the action mode of CS was updated according to recently published data to highlight the beneficial effects of using CS in ruminants.

## INTRODUCTION

The livestock-production industries are continuously working to increase animal production performance by increasing feed utilization efficiency with minimum wastage of nutrients such as greenhouse gases or undigested material [1]. The utilization of nutrients could be enhanced by reducing the production of wasteful gases during rumen fermentation or through hormonal modification [2]. Growth hormone is an important hormone that controls the growth rate in mammals. It is found in different animal tissues and organs, and its release is regulated by the growth hormone-releasing hormone (GHRH) [3]. Growth hormone could enhance the production performance of ruminants in different ways [4]. However, the direct use of growth hormone in animals has been banned in many countries due to the harmful effects of its metabolic residues on humans [5]. Stimulating the production of growth hormone in the body of animals could be another approach to enhance their production performance. Somatostatin is another crucial hormone competing with GHRH to inhibit growth hormone secretion [6]. Decreasing the production of somatostatin could increase growth hormone secretion, which ultimately enhances animal production performance.

Cysteamine (CS) is a bioactive molecule that has been tested orally or through the intravenous route and has been shown to have the potential to stimulate the production performance across a range of livestock species. CS significantly affects the somatotropic axis [7], controls the production of somatostatin, and protects the animals from oxidative stress [8]. CS increases the concentration of growth hormone by reducing somatostatin secretion [9]. Furthermore, CS enhances the production performance in animals not only by increasing the level of growth hormone and reducing somatostatin but also involved in increasing the activities of digestive enzymes [10], the plasma concentration of triiodothyronine (T3), thyroxine (T4), insulin, insulin-like growth factor-I and -II (IGF-I, IGF-II), gastrin, and prolactin in ruminants [11,12]. CS also significantly protects cells from oxidative damage via sulfhydryl-disulfide exchange reactions, which facilitate glutathione synthesis [13]. Previous studies proposed that CS could significantly improve feed efficiency by improving the apparent digestibility, weight gain, and milk production in various classes of ruminants [11,12,14]. Considering CS’s beneficial roles, it could be a novel growth promoter for the livestock industry. The present review article summarizes previous studies on using CS in different classes of ruminants and its mode of action.

## CHARACTERISTICS OF CS IN ANIMALS

### Physio-chemical properties of cysteamine

Cysteamine (also known as: β-mercaptoethylamine, 2-mercaptoethylamine, 2-aminoethanethiol, thioethanolamine, decarboxycysteine, and mercaptamine) having molecular formula HSCH_2_CH_2_NH_2_, is a solid, crystal compound with molecular weight 77.15 g/mol, with high water solubility (23.5 g/L). It is available as phospho-cysteamine, cysteamine-bitartrate, or cysteamine hydrochloride (CS-HCl), with the last form (CS-HCl) most commonly used as a feed additive in ruminants’ diet. Using CS in feed has some limitations, such as its bitter taste, sickening odor, hygroscopic nature, and chemical instability with poor pharmacokinetic profile. Due to these limitations, CS was used through an intravenous route to ensure the supply of a required amount of active compounds in the circulating system of animals. In addition, its pungent smell may negatively affect the feed palatability. However, using CS intravenously was not a feasible and practical approach for a large number of animals. With the advancement in encapsulation technology, many feed-grade compounds are being coated for their safe transfer and release at the site of action. Now, all undesirable properties of CS have been removed through encapsulation technology. Coated CS-HCl is also known as micro-encapsulated CS-HCl, with improved safety profile and pharmacokinetics by targeted release throughout the intestinal tract and with better effectiveness [15].

### Biosynthesis and metabolism of cysteamine

**Figure 1:**
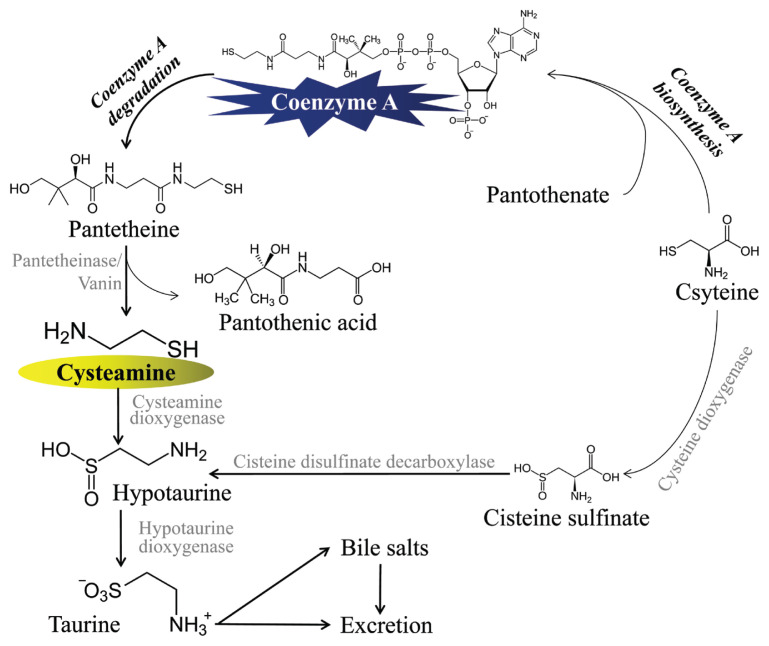
The biosynthesis and metabolism of cysteamine (CS).

CS exists in human milk [16] and is naturally produced inside the body of mammals through the breakdown of coenzyme A. Initially, coenzyme A is converted into pantetheine which is subsequently hydrolyzed to pantothenic acid and CS in the presence of pantetheinase (vanin; Figure 1). Coenzyme A could be produced through the reaction of cysteine and pantothenate. Regarding the degradation of CS, it could be oxidized non-enzymatically [17] or through enzymatic oxidation. During later oxidation, the thiol group of CS gets oxidized to hypotaurine (using 2-aminoethanethiol dioxygenase and cysteamine dioxygenase); subsequently, hypotaurine is converted into taurine [15], which is excreted through bile salts, urine, or feces [8]. Considering the welfare of animals, CS is safe to use because of its natural occurrence in milk and biosynthesis inside the body of mammals. In addition, the body can utilize and remove it, which suggests that CS is safe to use as a feed additive in livestock.

### Hormonal modifier

**Figure 2:**
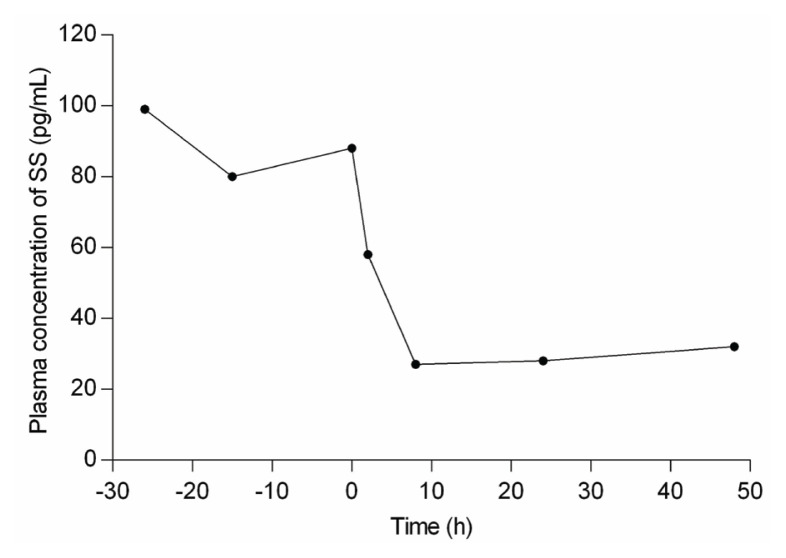
Effect of dietary supplementation of cysteamine (CS) (80 mg/kg body weight) on plasma concentration of somatostatin in sheep [14].

Somatostatin has an inhibitory effect on secretions from the gastrointestinal tract (GIT), pancreas, pituitary, endocrine, and exocrine systems; and has a role in modifying neurotransmission and memory formation in the central nervous system [18]. In ruminants, somatostatin negatively affects the flow rate of digesta, nutrient absorption, and metabolism, which affect their growth and development [9]. The critical role of CS is to regulate the somatostatin concentration in the circulatory system, hypothalamus, GIT, and abomasal tissue of animals [9,12]. Somatostatin works through paracrine and neuroendocrine actions. CS reduces the somatostatin concentration in these tissues and simultaneously reduces circulating somatostatin concentrations [9]. The somatostatin becomes activated by making disulfide bridges with cysteine residues that perform biological functions. CS does not allow somatostatin to make a disulfide bridge with cysteine residues, and its thiol group occupy this position by making mixed disulfide with the cysteine [7]. Secondly, somatostatin has a very short biological half-life; a study in rats showed rapid disappearance of somatostatin from circulation (*t*_1/2_ = 1 min), followed by slow clearance from skin and muscle [19]. At the same time, in humans, it is about *t*_1/2_ = 2–3 min [20]. It has been proposed that proteases are responsible for the rapid degradation of this hormone through catabolism [19]. Therefore, the presence of CS rapidly decreases the efficiency of somatostatin, as CS does not allow somatostatin to get activated [9]. It is also proposed that CS suppresses the synthesis and release of somatostatin from the hypothalamic region as it can cross the blood-brain barrier [21]. It has been observed that dietary supplementation of CS (80 mg/kg body weight [BW]) in sheep significantly reduced the plasma concentration of somatostatin within 2 h and reduced it to the lowest concentration (65% reduction) in 8 h and maintained it at this low level for about 48 h after administration of CS (Figure 2).

Use of CS in animals’ feed not only reduces the plasma concentration of somatostatin but also enhances the concentration of growth hormone, insulin, IGF-I, IGF-II [11,12], thyroid stimulating hormone (TSH), T_3_, T_4_, prolactin, and gastrin [9,22]. Increased concentrations of growth hormone, insulin and IGF-I have been reported in cattle using CS [12]. Collectively, CS modulated the hormonal profile to enhance the anabolic processes and reduce the catabolic activities. As CS enhances the production of digestive enzymes, supplementation of CS should be done carefully. High dosing of CS (>200 mg/kg BW) could develop duodenal ulcers due to enhanced secretion of gastric acid in response to higher serum gastrin levels [23].

### Antioxidative agent

CS works as an antioxidative agent through three different pathways: i) its thiol group serves as an antioxidant [24]; ii) it increases the concentrations of glutathione (GSH) in cells [25]; iii) it removes hydrogen peroxide and can also directly remove toxic products of lipid peroxidation and reactive oxygen species (ROS) [26,27]. GSH is the principal antioxidant agent at the cellular level, which protects the cells from oxidative damage, and its depletion leads to hepatocellular injury and fibrosis [27,28]. GSH is a peptide that is formed with three amino acids, glutamic acid, cysteine, and glycine. CS is the source of cysteine. During oxidative stress, the biosynthesis of CS is up-regulated by the degradation of coenzyme A, which provides the opportunity to use CS in feed to protect the cells from oxidative stress [29,30]. However, GSH cannot enter the cell readily; under the condition of intracellular GSH depletion, the intracellular concentration of GSH could be increased by using CS, which can cross cell membranes [31]. At this time, the thiol group of CS serves as an antioxidant to protect the cells from oxidative damage [24]; increases the concentrations of GSH in the cell, which get oxidized to glutathione disulfide to remove free radicals; boost intracellular transport of cysteine to promote GSH synthesis [25].

## APPLICATION OF CS IN RUMINANTS

### Effect of cysteamine on production performance in ruminants

In feeding experiments, dry matter intake (DMI), body weight gain (BWG), and feed conversion ratio (FCR) are referred to as essential parameters for determining the growth-promoting effect of any feed additive. Following previous studies, CS can be used as a feed additive because it can improve production performance in ruminants in different ways. Experiments exhibited that dietary supplementation (20 g/d) of 30% coated CS-HCl for 63 days could increase the BWG up to 15.68% in beef cattle [10], while a 54.17% increase in BWG was observed in yaks by using 30% coated CS-HCl at the level of 5 g/d for 28 days [32]. Similarly, a 16.03% increase in the BWG was observed in steers using 30% coated CS-HCl at 80 mg/kg BW for 56 days with a 15.24% reduction in FCR from the corresponding control group [33]. An increase in BWG up to 28.36% with a 24.43% reduction in FCR was observed in lambs by using ≥99% CS-HCl (80 mg/kg BW) for 35 days [14]. In another experiment of lambs, BWG was increased by 19.92% with a 13.51% reduction in FCR on using 50% coated CS at a dose rate of 60 mg/kg BW for 64 days [34]. Collectively, it could be stated that dietary supplementation of CS could increase BWG (beef cattle 15%; sheep 28%; yaks 54%; steers 16%; lambs 28%) and reduce FCR (13% to 24%) in different species of ruminants as shown in Table 1.

Milk production was increased by using CS in dairy cattle (7.6% by using 100 mg/kg BW CS [35]; 6.88% by using 40 g/d CS [36]; 5.01% by using 30% CS [37]; 7.14% by using 30 g/d of 20% CS-HCl for 63 days [38]). CS dietary supplementation could increase milk yield (5% to 7%) and milk protein (2% to 8%) in Holstein, as shown in Table 1.

CS also has the potential to enhance wool production from 8% (using 80 mg/kg BW of ≥99% CS-HCl for 35 days [14]) to 62% (using 50 mg/kg BW CS for 120 days [11]) in lambs (Table 1). Another study proposed that CS increased the length of wool fiber (24.04%) and diameter (217.30%) by using 350 mg/kg BW CS for 120 days [39].

Dietary supplementation of CS enhances the expression of ghrelin mRNA [40], which could boost animal appetite and promote gastric acid secretion [41]. Other studies CS showed that it could enhance the digestibility of crude protein, crude fat, neutral detergent fiber, and gross energy in cattle. 8.31% and 9.44% increase in digestibility of crude protein and ether extract, respectively, were observed in beef cattle by using 30% coated CS-HCl (20 g/d) for 63 days [10], while digestibility of crude protein (1.97%), neutral detergent fiber (1.07%) and gross energy (11%) were enhanced in Holstein by using 30% coated CS-HCl (15 g/d) for 56 days [42] (Table 1). Enhanced gastric acid secretion might be the reason for improved digestibility of different nutrients, which boosts animal growth performance. Experiments suggested that CS enhances the efficiency of nutrients utilization as BWG was enhanced with decreased DMI (2.77%) in steers (using 30% coated CS-HCl at a level of 80 mg/kg BW for 56 days [33]) and lambs (using 80 mg/kg BW of ≥99% CS-HCl for 35 days [14]). CS itself helps to improve the productivity of animals. In supporting these results, another experiment suggested that CS changed glucolipid metabolism by enhancing plasma glucose, non-esterified fatty acids (NEFA), and lactate to their peak levels within the interval of 2 h when administered via the intravenous route (50 mg/kg BW of CS-HCl; Sigma Chemical, St. Louis, MO, USA) [9].

These findings supported that CS reduced the concentration of somatostatin having inhibitor effects on the functioning of gastrointestinal tissue, reducing digestion and absorption. CS not only enhanced the milk production efficiency but also enhanced the milk protein yield in dairy animals [37,43]. Possible reason for increasing milk protein might be increased microbial crude protein (MCP) synthesis during the rumen fermentation process, as stated by Wang et al [44] that CS could increase the MCP up to 22.61%, ammonia nitrogen (NH_3_-N) by 18.13%, and volatile fatty acids (VFAs) by 18.86% in goats when fed on 15 mg/kg BW of coated CS-HCl for eight days (Table 2). Another possibility of enhanced milk protein might be increased growth hormone production under the influence of CS, as increased growth hormone-enhanced insulin secretion (30%) [37] and portal blood flow [45]. Increased insulin production and enhanced blood flow could increase the uptake of glucose and amino acids by mammary gland leading to increased milk production, milk protein and fat synthesis [46].

### Effect of cysteamine on rumen fermentation and microbiome

An optimal pH (6.2 to 7.0) is essentially required for a typical ruminal environment [47], and dietary supplementation of coated CS had no effect on rumen pH, and it remained within the normal range [34]. The NH_3_-N is produced during rumen fermentation because it is required to synthesize MCP. A study on the use of CS (50% coated CS) showed that it could enhance the NH_3_-N (15.79%), MCP (7.43%), propionic acid (12.23%), and total VFAs (15.64%) in lambs when fed for 64 days at the levels of 60 mg/kg BW [34]. Enhanced VFAs production in goats by 11.23% (using 120 mg/kg BW of 30% coated CS-HCl for 40 days [48]) to 18.86% (using coated CS-HCl 15 mg/kg BW for eight days in goats, [44]) and in dairy buffalo 14.61% (using 50 g/d of 20% CS for 42 days [49]) was also observed by using CS, indicating that CS could improve the rumen fermentation efficiency. VFAs are the primary energy source for ruminants, which fulfill 75% of their digestible energy requirements and have a significant role in different metabolic activities [50]. Incubation of rumen fluid with CS also enhances VFAs production *in vitro* [51]. Propionic acid is one of the VFAs, with gluconeogenic properties that significantly contribute to glucose synthesis in ruminants [50]. CS has been reported to enhance the production of propionic acid during rumen fermentation in buffalo by 33.64% (using 50 g/d of 20% CS for 42 days [49]), in lambs by 12.23% (using 60 mg/kg BW of 50% coated CS-HCl for 64 days [34]), and also in an *in vitro* study [51]. However, contrary results were also observed using CS in goats [44,48]. Results of these studies suggested that CS changed the rumen fermentation pattern to enhance the production of glucogenic propionate, which ultimately enhanced energy utilization efficiency. According to the aforementioned information, dietary supplementation of coated CS showed a positive effect on rumen fermentation, which could be the reason for enhanced production performance. It has been proposed that CS modifies the secretion of hormones involved in smooth muscle contraction and relaxation, as dietary supplementation of 30% coated CS-HCl (20 g/d) for 63 days increases the secretion of T_3_ up to 26.52% than the control group [10]. Elevated T_3_ level reduces the mean retention time (MRT) of digesta in ruminants [52]. A change in MRT affects the fermentation process, which enhances the microbial growth rate and higher nitrogen flow to the duodenum, as reported in sheep by using CS [53]. On the other hand, *in vitro* study showed that CS decreased ammonia production and enhanced VFAs in rumen fluid [51], which suggested a direct effect of CS on the rumen fermentation process rather than just modifying the MRT. In addition, McLeod et al [9] proposed that CS boost the net absorption of intestinally available nutrients to their peak levels (plasma glucose 10.71 mM, NEFA 1.26 mM, lactate 6.37 mM) within two hours after intravenous administration of CS (50 mg/kg BW of CS-HCl; Sigma Chemical) which return to pre-treatments levels (glucose 3.66 mM, NEFA 0.23 mM, lactate 1.12 mM) by 8 h for glucose and 24 h for other nutrients. CS tries to modify the fermentation process in a way beneficial for host animals, enhance intestinal digestion, and subsequently increase the efficiency of absorbing these nutrients, which are collectively linked to the improved animal production performance.

Methane (CH_4_) is a potent kind of greenhouse gas that is a crucial contributor to global warming, and its production during rumen fermentation could reduce dietary energy availability to animals by up to 12% [54]. Previous studies also proved that CS could reduce the CH_4_ production in steers and lambs [14,33,34]. CS could reduce CH_4_ production up to 9.20% in steers [33] and 11.40% in lambs [14] at a dietary supplementation level of 80 mg/kg BW, the difference in CH_4_ reduction might be due to the difference in purity of CS used (≥99% CS-HCl vs 30% CS-HCl), experimental duration or different species. Another experiment on lambs showed a 12.57% reduction in CH_4_ production by using 60 mg/kg BW of 50% coated CS-HCl for 64 days [34]. These results suggested that CH_4_ production could be reduced from 9% to 12% in ruminants (Table 2). There are likely two mechanisms through which CS could work to reduce CH_4_ production. i) Direct effect: reduction in methanogenic microorganisms, about 75% reduction in protozoa population was reported during *in vitro* analysis with CS [33]. CS has been reported to stimulate the production of fatty acids [55], and unsaturated fatty acids, which are toxic to protozoa [56]. Long-chain fatty acids produced in response to CS could make the food particles unavailable to the protozoa and reduce their population. In addition, long-chain unsaturated fatty acids could affect the protozoal protein content and enhance the dehydrogenase activity [57]. All these conditions collectively cause a reduction in the population of protozoa. ii) Indirect effect: propionic acid production consumed H_2_, while acetic acid production released H_2_ [52], so enhanced propionic acid production with CS could reduce CH_4_ production.

The health status and metabolic activities in ruminants are also dependent on the diversity of rumen microbiota [58,59]. Previous studies suggested that CS did not affect bacterial and fungal alpha diversity indices in goats fed 120 mg/kg BW of 30% coated CS-HCl for 40 days [48]. Dietary supplementation of CS supports the dominance of *Bacteroidetes* and *Firmicutes* (bacterial phyla), and *Neocallimastigomycota* and *Ascomycota* (fungal phyla) in buffalo (fed 20% CS at level of 50 g/d for 42 days [49]) and goats (using 120 mg/kg BW of 30% coated CS-HCl for 40 days [48]), respectively. Both phyla are important because these microbes are involved in the degradation and fermentation of dietary nutrients. Microbial analysis at the genus level showed that *Prevotella-1* was the dominant bacteria in goats fed 120 mg/kg BW of 30% coated CS-HCl for 40 days [48]. Bacteria of this genus play a significant role in the degradation and utilization of starch, pectin, hemicellulose, proteins, and peptides [60]. *Piromyces* was the dominant genus of fungi in response to CS supplementation in cashmere goats (fed 120 mg/kg BW of 30% coated CS-HCl for 40 days [48]). A significant increase in the abundance of *Weissella* and *Lactococcus* was also observed by using CS in cashmere goats (fed 120 mg/kg BW of 30% coated CS-HCl for 40 days [48]). These bacteria have antioxidant activity [61,62] and probiotic potential [63], as they are associated with lactic acid bacteria. Relative abundance of Bacteroidetes genera (*Tannerella*, *Alloprevotella*, and *U29-B03*) involved in carbohydrate degradation, protein hydrolysis, and fermentation of amino acids [64] were increased with CS supplementation in cashmere goats (fed 120 mg/kg BW of 30% coated CS-HCl for 40 days [48]) and growth-retarded yaks (fed 30% coated CS-HCl at level of 5 g/d for 28 days [32]). Based on these results, it could be stated that CS increases the availability of nutrients to the animals and enhances the efficiency of nutrient absorption and fermentation capacity in ruminants.

### Effect of cysteamine on hormones and blood metabolites in ruminants

Animal growth and development depend on different factors including age, genetic potential, neuroendocrine function, and metabolism. From the endocrine system, growth hormone and IGF-I mainly regulate animal growth performance. Somatostatin is a peptide hormone secreted by the hypothalamus, inhibiting the secretion of growth hormone from somatotrophs [65]. Most of the studies on different ruminants (dairy cattle [38]; beef cattle [10]; goats [48]; lambs [34]) showed a significant reduction in somatostatin by using CS, suggesting the inhibitory effect of CS on somatostatin. Reduction in somatostatin secretion has been observed from 13.30% (using coated CS-HCl 15 mg/kg BW for eight days in goats [44]) to 88.40% (using 100 mg/kg BW CS in Holstein [35]) by dietary supplementation of CS. CS, with a reducing agent, the thiol group, breaks the disulfide bond in the molecular structure of somatostatin [34]. The anterior pituitary gland secretes growth hormone has a critical role in somatic growth, and responsible for the secretion of IGF-I from the liver [66]. An increase in growth hormone has been observed from 29% (using 30% coated CS-HCl at a level of 5 g/d for 28 days in yaks [32]) to 57.42% (using 60 mg/kg BW of 50% coated CS-HCl for 64 days in lambs [34]) as compared to corresponding control groups in ruminants. In addition, CS could also enhance the production of other growth-stimulating hormones, including insulin [10,37], IGF-I [10,48,67], T_3_ and T_4_ [10,68] in animals. It is proposed that CS reduces the inhibitory effect of somatostatin on thyroid-stimulating hormone, which increase the concentration of GH, leading to enhanced concentration of T_3_ [69]. Somatostatin has inhibitory effects on pituitary, thyroid, and digestive functions, and suppression of somatostatin by CS could enhance nutrient utilization and stimulate animal growth performance. Table 3 summarizes different studies which showed that varied dietary supplementation of CS in different forms could increase the production of growth hormone by 29% to 57%, IGF-1 by 23% to 60%, and insulin by 19% to 30% and reduce the concentration of somatostatin by 13% to 88% in ruminants.

CS could have a productive role in mammary gland development which is strictly controlled by hormones i.e., estrogen, progesterone, prolactin, and growth factor [70,71]. IGF-I is an important growth factor that regulates all hormones required for mammary gland development, especially for ductal growth of mammary glands during the pubertal period. In addition, postnatal effects of growth hormone on mammary glands development are mediated principally by IGF-I [66,72,73], and the presence of IGF-I is also required for the action of estrogen and progesterone [74]. These findings highlighted the key value of IGF-I in mammary glands development [72] and dietary supplementation of CS could enhance the concentration of IGF-I by 23% to 60% (Table 3), which is indirectly linked with improvement in mammary glands development, which could be the reason of enhanced milk production in dairy animals.

CS could alter the concentration of different blood metabolites in a way beneficial for the growth performance of the animals. A reduced concentration of circulating NEFA observed by using CS in cows might be due to the increase uptake of NEFA by mammary glands under the influence of CS, which increase the milk fat yield by 2.01% (using 30 g/d of 20% CS-HCl for 63 days [38]). Reduced concentration of circulating NEFA is also beneficial for animals as it reduces the risk of ketosis. Consistent with these findings reduced incidence of mastitis was also declared by using CS in another study [43]. CS also reduced the blood urea nitrogen (5.26%) in cows (using 30 g/d of 20% CS-HCl for 63 days [38]), which might be the reason for enhanced nitrogen utilization efficiency and increased milk production in dairy cows.

### Effect of cysteamine on antioxidant potential in ruminants

**Figure 3:**
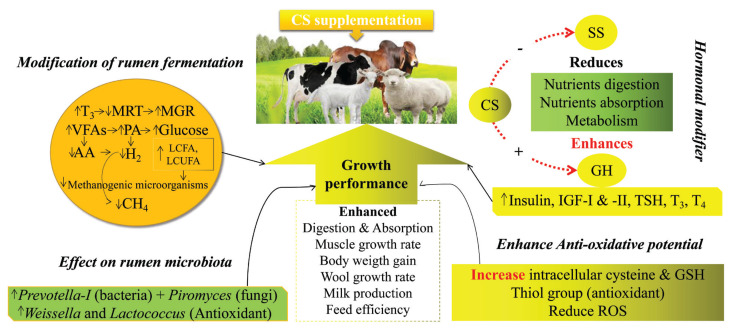
Mode of action of cysteamine to enhance the production performance in ruminants. AA, acetate; CS, cysteamine; FA, fatty acid; GH, growth hormone; GSH, glutathione; H_2_, hydrogen gas; IGF-I & -II, insulin-like growth factor-I and insulin-like growth factor-II; LCFA, long chain fatty acid; LCUFA, long chain unsaturated fatty acid; MGR, microbial growth rate; MRT, mean retention time; PA, propionic acid; ROS, reactive oxygen species; SS, somatostatin; T_3_, triiodothyronine; T_4_, thyroxine; TSH, thyroid stimulating hormone; VFAs, volatile fatty acids.

CS has a vital role in removing oxidative stress by increasing the concentration of GSH in ruminants. An increase of 19.6% GSH was observed in Holstein (fed 15 g/d of 30% coated CS-HCl for 56 days [42]), 20.64% in lambs (fed 50 mg/kg BW of ≥99% CS-HCl [68]), and 26.57% in goats (fed 120 mg/kg BW of 30% coated CS-HCl for 40 days [48]). At the same time, CS could reduce the levels of malondialdehyde (MAD), which was reduced to 26.77% in lambs (fed 50 mg/kg BW of ≥99% CS-HCl [68]) and 2.64% in goats (fed 120 mg/kg BW of 30% coated CS-HCl for 40 days [48]) using CS. MAD is a biochemical marker of oxidative stress which is used as a procurator for lipid peroxidation and cellular damage *in vivo* [75,76]. Results of a basic experiment conducted on rats showed that CS could reduce the levels of ROS, MAD and increase the activities of GSH in their brain cortex after 48 h of administration of CS [77]. CS reduces oxidative stress and protects cells from oxidative damage, which could be another reason for improved growth performance. The antioxidant capacity of CS also protects the developing embryo from oxidative stress during *in vitro* study [78]. CS enhances the concentration of GSH, which is essentially required to protect the embryo from oxidative stress until it becomes able to synthesize its own GSH. Collectively, dietary supplementation of coated CS at varied levels could increase the level of GSH from 19% to 26% and reduce MDA by 2% to 26% in ruminants (Table 3). CS does not protect the cells from oxidative stress at a very high dose rate because at high doses (23 to 91 μmol/L CS) it could produce H_2_O_2_ [79]. Secondly, at higher doses, it could decrease the activities of glutathione peroxidase, which is required for proper functioning of GSH [78]. Altogether, a detailed mode of actions of CS in ruminants to enhance their production performance is shown in Figure 3.

## CONCLUSION

Available data on the use of CS in ruminants suggested that protected CS, e.g., the microencapsulated CS-HCl, could be a safe feed additive for ruminants because of its potential to improve meat, milk, and wool production. Secondly it is a biological compound, so there is no objection to its use in the feed of ruminants. CS offers advantages by enhancing the anabolic activities through somatotropic axis stimulation and rumen fermentation pattern modification. However, there is a need to determine the effect of using CS on meat quality, udder development, and long-term reproductive performance in ruminants for healthy breeding of the ruminant industry.

## Figures and Tables

**Table 1:** Effect of cysteamine on different parameters of production performance in ruminants

Reference	Type and purity	Animal	Dose (mg/kg BW)	Duration (d)	Percentage change in CS-treated animals relative to control				
Growth performance					DMI	Weight gain		Feed conversion ratio	
[10]	30% coated CS-HCl	Beef cattle	20 g/d	63		15.68			
[12]	NA	Beef cattle	50	63	1.3	15.90			
[14]	≥ 99% CS-HCl	Lambs	80	35	−2.45	28.26		−24.43	
[32]	30% coated CS-HCl	Yaks	5 g/d	28		54.17			
[33]	30% CS-HCl	Steers	80	56	−2.77	16.03		−15.24	
[34]	50% coated	Lambs	60	64	3.76	19.92		−13.51	
					
Milk production and milk quality					Milk yield	Milk protein		FCM	DMI
				
[35]	NA	Holstein	100	NA	7.6				
[36]	NA	Holstein	40 g/d	NA	6.88	2.76		6.56 (3%)	
[37]	30%	Holstein	30 g/d	NA	5.01			6.67 (4%)	2.34
[38]	20% CS-HCl	Holstein	30 g/d	63	7.14	8.71			
					
Wool growth					Wool growth rate	Gain in length of wool		Gain in diameter of wool	ADG
				
[11]	NA	Lambs	50	120	62				17.9
[14]	≥99% CS-HCl	Lambs	80	35	8				
[39]	NA	Lambs	350	120		24.04		217.30	12.77
					
Apparent digestibility of nutrients					CP	EE	NDF	ADF	GE
				
[10]	30% coated CS-HCl	Beef cattle	20 g/d	63	8.31	9.44			
[42]	30% coated CS-HCl	Holstein	15 g/d	56	1.97		1.07	−1.53	11

BW, body weight; CS, cysteamine; DMI, dry matter intake; NA, information not available; FCM, fat corrected milk; ADG, average daily gain; CP, crude protein; EE, ether extract; NDF, neutral detergent fiber; ADF, acid detergent fiber; GE, gross energy.

**Table 2:** Effect of cysteamine on rumen fermentation parameters in ruminants

Reference	Type and purity	Animal	Dose (mg/kg BW)	Duration (d)	Percentage change in CS-treated animals relative to control					

					Methane	NH_3_-N	AA	PA	BA	VFAs
[14]	≥99% CS-HCl	Lambs	80	35	−11.40					
[33]	30% CS-HCl	Steers	80	56	−9.20					
[34]	50% coated	Lambs	60	64	−12.57	15.79	−4.78	12.23		15.64
[44]	Coated CS-HCl	Goats	15	8		18.13	2.05	−2.4	5.46	18.86
[48]	30% coated CS-HCl	Goats	120	40		37.50	3.04	−5.70	−7.91	11.23
[49]	20%	Buffalo	50g/d	42			7.80	33.64	38.99	14.61

BW, body weight; AA, acetate; PA, propionic acid; BA, butyric acid, VFAs, total volatile fatty acids.

**Table 3:** Effect of cysteamine on different hormones and blood metabolites in ruminants

Reference	Type and purity	Animal	Dose (mg/kg BW)	Duration (d)	Percentage change in CS-treated animals relative to control					
Hormones					Growth hormone	IGF-I	Insulin	Somatostatin	T_3_	T_4_
				
[10]	30% coated CS-HCl	Beef cattle	20 g/d	63	48.00	60.84	30.17	−21.59	26.52	19.14
[32]	30% coated CS-HCl	Yaks	5 g/d	28	29.00			−16		
[34]	50% coated	Lambs	60	64	57.42	23.77		−13.89		
[35]	NA	Holstein	100	NA	33.10			−88.4		
[37]	30%	Holstein	30 g/d	NA			19.82			
[38]	20% CS-HCl	Holstein	30 g/d	63	50.52			−54.07		
[44]	Coated CS-HCl	Goats	15	8	29.38			−13.30		
[48]	30% coated CS-HCl	Goats	120	40	28.82	31.45	4.29	−30.01		
					
Blood metabolites					GSH		BUN		MDA	
				
[38]	20% CS-HCl	Holstein	30 g/d	63			−5.26			
[42]	30% coated CS-HCl	Holstein	15 g/d	56	19.6					
[48]	30% coated CS-HCl	Goats	120	40	26.57				−2.64	
[68]	≥99% CS-HCl	Lambs	50*	30	20.64				−26.77	

* Intravenous application of CS on 1st, 10th, and 20th days of the experiment through the jugular vein. NA, information not available.

BW, body weight; CS, cysteamine; IGF-I, insulin-like growth factor-I; T_3_, triiodothyronine; T_4_, thyroxine; GSH, glutathione; BUN, blood urea nitrogen; MDA, malondialdehyde.
